# A first-in-human phase 1/2 study of FGF401 and combination of FGF401 with spartalizumab in patients with hepatocellular carcinoma or biomarker-selected solid tumors

**DOI:** 10.1186/s13046-022-02383-5

**Published:** 2022-06-02

**Authors:** Stephen L. Chan, Martin Schuler, Yoon-Koo Kang, Chia-Jui Yen, Julien Edeline, Su Pin Choo, Chia-Chi Lin, Takuji Okusaka, Karl-Heinz Weiss, Teresa Macarulla, Stéphane Cattan, Jean-Frederic Blanc, Kyung-Hun Lee, Michela Maur, Shubham Pant, Masatoshi Kudo, Eric Assenat, Andrew X. Zhu, Thomas Yau, Ho Yeong Lim, Jordi Bruix, Andreas Geier, Carmen Guillén-Ponce, Angelica Fasolo, Richard S. Finn, Jia Fan, Arndt Vogel, Shukui Qin, Markus Riester, Vasiliki Katsanou, Monica Chaudhari, Tomoyuki Kakizume, Yi Gu, Diana Graus Porta, Andrea Myers, Jean-Pierre Delord

**Affiliations:** 1 State Key Laboratory of Translational Oncology, Department of Clinical Oncology, Sir YK Pao Centre for Cancer, The Chinese University of Hong Kong, Hong Kong, China; 2 West German Cancer Center, University Hospital Essen, Germany & German Cancer Consortium (DKTK), Partner site University Hospital Essen, Essen, Germany; 3 Asan Medical Center, University of Ulsan College of Medicine, Seoul, South Korea; 4 Department of Oncology, National Cheng Kung University Hospital, College of Medicine, National Cheng Kung University, Tainan, Taiwan; 5 Centre Eugène Marquis, Rennes, France and ARPEGO (Accès à La Recherche Précoce Dans Le Grand-Ouest) Network, Rennes, France; 6 National Cancer Centre, Singapore, Singapore; 7 National Taiwan University Hospital, Taipei, Taiwan; 8 National Cancer Centre Hospital, Tokyo, Japan; 9 Salem Medical Center, Internal Medicine, Heidelberg, Germany; 10 Vall d’Hebron University Hospital, Vall d’Hebron Institute of Oncology (VHIO), IOB Quirón, Barcelona, Spain; 11 Chru de Lille, Lille, France; 12 CHU de Bordeaux Pessac, Bordeaux, France; 13 Seoul National University Hospital, Seoul, South Korea; 14 University Hospital of Modena, Modena, Italy; 15 MD Anderson Cancer Center, Houston, TX USA; 16 Kindai University Hospital, Osaka, Japan; 17 Hôpital Saint-Eloi Montpellier, Montpellier, France; 18 Massachusetts General Hospital, Boston, MA USA; 19 Jiahui International Cancer Center, Jiahui Health, Shanghai, China; 20 Queen Mary Hospital, Hong Kong, China; 21 Samsung Medical Center, Seoul, South Korea; 22 Barcelona clinic liver cancer (BCLC) Group, Liver Unit, Hospital Clínic, IDIBAPS, CIBERehd, University of Barcelona, Barcelona, Spain; 23 University Hospital Würzburg, Würzburg, Germany; 24 Hospital Universitario Ramon y Cajal, IRYCIS, Madrid, Spain; 25 San Raffaele Hospital, Milan, Italy; 26 University of California, Los Angeles, California, USA; 27 Zhongshan Hospital, Fudan University, Shanghai, China; 28 Hannover Medical School, Hanover, Germany; 29 No. 81th PLA Hospital Nanjing, Jiangsu, China; 30 Novartis Institutes for BioMedical Research, Cambridge, MA USA; 31 Novartis Institutes for BioMedical Research, Basel, Switzerland; 32 IQVIA, Durham, North Carolina, USA; 33 Novartis Pharma K.K, Tokyo, Japan; 34 Novartis Pharmaceuticals Corporation, East Hanover, NJ USA; 35 IUCT Oncopole - Institut Claudius Regaud, Toulouse, France

**Keywords:** Hepatocellular carcinoma, FGFR4, PD-1, PD-L1, Immune checkpoint inhibitors, Phase 1, KLB, FGF19

## Abstract

**Background:**

Deregulation of FGF19-FGFR4 signaling is found in several cancers, including hepatocellular carcinoma (HCC), nominating it for therapeutic targeting. FGF401 is a potent, selective FGFR4 inhibitor with antitumor activity in preclinical models. This study was designed to determine the recommended phase 2 dose (RP2D), characterize PK/PD, and evaluate the safety and efficacy of FGF401 alone and combined with the anti-PD-1 antibody, spartalizumab.

**Methods:**

Patients with HCC or other FGFR4/KLB expressing tumors were enrolled. Dose-escalation was guided by a Bayesian model. Phase 2 dose-expansion enrolled patients with HCC from Asian countries (group1), non-Asian countries (group2), and patients with other solid tumors expressing FGFR4 and KLB (group3). FGF401 and spartalizumab combination was evaluated in patients with HCC.

**Results:**

Seventy-four patients were treated in the phase I with single-agent FGF401 at 50 to 150 mg. FGF401 displayed favorable PK characteristics and no food effect when dosed with low-fat meals. The RP2D was established as 120 mg qd. Six of 70 patients experienced grade 3 dose-limiting toxicities: increase in transaminases (*n* = 4) or blood bilirubin (*n* = 2). In phase 2, 30 patients in group 1, 36 in group 2, and 20 in group 3 received FGF401. In total, 8 patients experienced objective responses (1 CR, 7 PR; 4 each in phase I and phase II, respectively). Frequent adverse events (AEs) were diarrhea (73.8%), increased AST (47.5%), and ALT (43.8%). Increase in levels of C4, total bile acid, and circulating FGF19, confirmed effective FGFR4 inhibition.

Twelve patients received FGF401 plus spartalizumab. RP2D was established as FGF401 120 mg qd and spartalizumab 300 mg Q3W; 2 patients reported PR.

**Conclusions:**

At biologically active doses, FGF401 alone or combined with spartalizumab was safe in patients with FGFR4/KLB-positive tumors including HCC. Preliminary clinical efficacy was observed. Further clinical evaluation of FGF401 using a refined biomarker strategy is warranted.

**Trial registration:**

NCT02325739.

**Supplementary Information:**

The online version contains supplementary material available at 10.1186/s13046-022-02383-5.

## Background

Hepatocellular carcinoma (HCC) is the sixth most common cancer worldwide and the fourth most common cause of cancer-related deaths [1, 2]. Most patients present with unresectable progressive disease with approximate survival of one year [3]. Systemic therapy with first-line multikinase inhibitors such as sorafenib and lenvatinib have been the standard of care [4], but have considerable side effects impacting quality of life [5, 6]. Recent advances in the development of PD-1 and PDL-1 inhibitors, such as pembrolizumab and nivolumab, alone [7, 8] and in combination with targeted agents [9, 10] have provided additional options for patients with HCC [11]. Many such combinations like atezolizumab-bevacizumab have been approved by US FDA [12]. Despite this, overall survival in advanced disease remains poor and there remains a need for improved therapies for patients with HCC.

Fibroblast growth factor 19 (FGF19)/fibroblast growth factor receptor 4 (FGFR4) signals play an important role in hepatobiliary physiology [13–17]. Induced in the ileum in response to the release of bile acids upon food ingestion, FGF19 circulates to the liver where its receptor FGFR4 and the co-receptor B-Klotho (KLB) are co-expressed, to suppress CYP7A1, the rate-limiting enzyme for bile acid synthesis and thus, limiting bile acid release into the intestine [18]. Various preclinical reports and a recent clinical study suggest that the FGF19-FGFR4 signaling network is an oncogenic driver of certain forms of HCC and other solid malignancies with aberrant FGF19 expression [14, 15, 19]. In addition, FGF19 expressed by nontumor cells has been shown to induce hepatocyte proliferation and dysplastic changes throughout the hepatic lobule ultimately resulting in HCC [20].

FGF401 (roblitinib) is a reversible, covalent, potent and highly selective FGFR4 inhibitor with antitumor activity in FGF19/FGFR4-dependent tumor models [21–23]. It inhibits growth of HCC and gastric cancer cell lines expressing FGFR4, KLB and FGF19 with excellent selectivity over non-sensitive tumor models. In cell-line derived HCC xenografts and patient-derived HCC xenografts, FGF401 robustly induces regression/stasis in a dose-dependent manner. Considering the supportive preclinical data, FGF401 provides an opportunity to target solid tumors, specifically FGF19/FGFR4–dependent HCC.

In a recent study, lenvatinib reduced the tumor PD-L1 level and Treg differentiation to improve anti-PD-1 efficacy by blocking FGFR4 [24]. To further explore the combination opportunity of PD-1 inhibitors with FGFR4 inhibition, spartalizumab (PDR001), a humanized immunoglobulin G4κ monoclonal antibody that binds PD-1 and blocks its interaction with PD-L1/PD-L2 [25] was also explored along with FGF401.

We conducted a first-in-human clinical trial with single-agent FGF401 or in combination with spartalizumab, administered to patients with HCC or other solid malignancies. The key objective of the study was to determine the maximum tolerated dose (MTD) and/or recommended phase 2 dose (RP2D) of FGF401 as a single agent or in combination with spartalizumab. We further evaluated the pharmacokinetic and pharmacodynamic characteristics, safety, and efficacy of the 2 treatment regimens.

As the prevalence of HCC is much higher in Asia-Pacific region and other etiological factors could differ across geographic regions [26], the dose expansion was stratified into Asian and non-Asian population.

## Methods

### Study oversight

This proof-of-concept phase 1/2 clinical study was conducted in accordance with the International Conference on Harmonization E6 Guidelines for Good Clinical Practice, applicable regulations, and the principles of the Declaration of Helsinki. The study protocol and all amendments were approved by the independent ethics committee or institutional review board at each study site. All patients provided written informed consent before any study-specific procedure was performed. The study had a phase 1 dose-escalation part followed by a phase 2 dose-expansion part, started in December 2014 and ended by May 2019 (NCT02325739).

### Patients

We enrolled adult patients with progressive HCC or other solid malignancies and Eastern cooperative oncology group (ECOG) performance ≤1 from 27 sites across 11 countries or regions (China, France, Germany, Hong Kong, Italy, Japan, Korea, Singapore, Spain, Taiwan, and USA). HCC diagnosis was as per AASLD guidelines and patients with BCLC stage C and Child-Pugh class A (5–6 points) with no encephalopathy and/or ascites were eligible. Following initial enrollment, patients were assigned to the single-agent FGF401 arm in phase 1 (dose escalation) and later in phase 2 (dose expansion). Upon the completion of phase 1 and while phase 2 was ongoing, patients were recruited to phase 1 of the combination arm (FGF401 and spartalizumab). Subsequent enrolment of patients in phase 2 of the combination arm was not initiated.

In the phase 1 part of FGF401 single agent, patients with HCC or other advanced solid tumors characterized by positive FGFR4 and KLB transcript expression were enrolled. The expression was assessed in the pre-treatment tumor biopsies obtained during molecular prescreening by means of RT-qPCR. Biomarker positivity was defined by a Novartis-designated laboratory that was certified to perform clinical assays. FGF19 mRNA expression was also assessed by RT-qPCR, but it was not used as an inclusion criterion. Owing to the fact that most of the patients with HCC were positive for FGFR4 and KLB, during the phase 2 part of the FGF401 single agent, evidence of positive expression was required in order to begin screening activities only for patients in group 3. Molecular prescreening was not performed for the FGF401 and spartalizumab combination part.

Phase 1 of the single-agent arm comprising patients whose tumors were positive for FGFR4 and KLB received treatment under either fasted (stratum 1) or fed (stratum 2: food-effect cohort) conditions. In phase 2, additional patients were enrolled in 3 groups: groups 1 and 2 enrolled patients with HCC from Asian and non-Asian regions, respectively, who had prior systemic treatment with sorafenib with documented intolerance or disease progression during or after its discontinuation and group 3 enrolled double positive progressive patients with other solid malignancies regardless of the geography.

Phase 1 of the combination arm comprised patients who had received up to 2 previous lines of systemic treatment including sorafenib with documented intolerance or disease progression during or after its discontinuation. Patients with previous treatment with any FGF19-FGR4 inhibitor or who discontinued prior anti-PD-1/PD-L1 therapy due to an anti PD-1/PD-L1–related toxicity were excluded.

### Treatment regimen

**Fig. 1:**
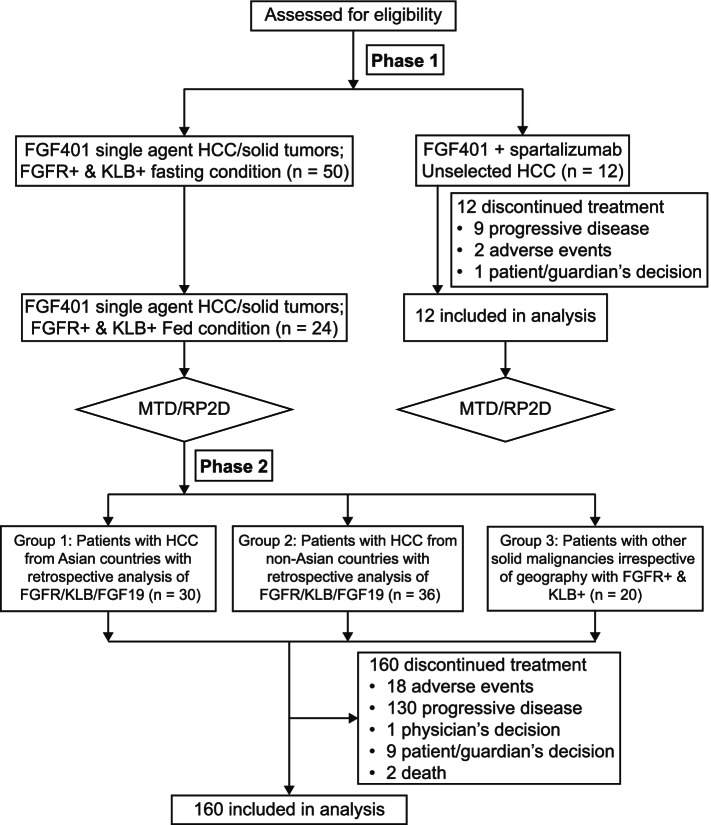
Study design

FGF401 was administered per oral under fasted condition or with low-fat meal on a continuous once daily (qd) regimen for both FGF401 single agent and in combination with spartalizumab. A 300-mg intravenous infusion of spartalizumab was administered once every three weeks (q3w) and 1 treatment cycle was defined as 21 days. The study treatment was administered until the patient experienced unacceptable toxicity, progressive disease and/or the treatment was discontinued at investigator’s discretion or patient’s withdrawal of consent. Figure 1 summarizes the study design and visit flow.

#### FGF401 single-agent arm

Phase 1: Escalation with overdose control (EWOC) principle based on the observed dose-limiting toxicities (DLTs) was employed while escalating dosage starting with 50 mg until the lowest of MTD and/or RP2D was met. Sample size to have reasonable operating characteristics was estimated to be a minimum of 21 evaluable patients.

To further assess the effect of food uptake on FGF401, additional patients were recruited upon observance of clinical activity to receive doses under fed condition, in parallel with the fasted dose escalation. The starting dose was estimated at 80 mg qd. Principles of dose escalation aligned with those under fasted condition.

Phase 2: As part of the dose expansion, additional patients with advanced HCC or other solid tumors were enrolled into 3 groups at the RP2D: groups 1 and 2 comprised patients with HCC from Asian and non-Asian countries, respectively and group 3 comprised double-positive patients with other solid malignancies regardless of the geography. The former 2 groups were recruited regardless of their double positivity, with the aim of analyzing it retrospectively.

#### Combination arm (FGF401 with spartalizumab)

Phase 1: Cohorts of eligible patients were treated with increasing doses of FGF401 in combination with a fixed dose of spartalizumab until the MTD and/or RP2D was met. For each untested dose level, the administration of the first dose of the study treatment was staggered by 24 hours for the first 3 patients. The dose escalation was guided by the EWOC principle. A minimum of 12 patients were required to have reasonable operating characteristics to determine the MTD of the combination treatment.

### Assessments

The primary endpoint for the phase 1 parts was the incidence of DLTs evaluated. The probability of DLTs during the evaluation period was estimated for patients in the dose determining set (DDS) for each treatment arm, FGF401 single agent and FGF401 + spartalizumab combination, to estimate respective MTDs. DDS for the phase 1 single agent or combination comprised safety set patients who either experienced a DLT or had confirmation by both the sponsor and investigators that no DLT occurred during the first cycle, C1 (first 2 cycles: C1, C2) based on at least 66% of planned doses administered in C1 (C1 and C2) with a minimum of 21 (42) days of observation following the first dose. For phase 2, the primary efficacy endpoint to investigate the antitumor activity of FGF401 included time to tumor progression assessed in groups 1 and 2, and overall response rate (ORR) in group 3 based on local assessment per Response Evaluation Criteria in Solid Tumors (RECIST) v1.1.

The secondary endpoints characterizing safety, efficacy, pharmacokinetics (PK), immunogenicity, and food effect properties of the FGF401 and the combination were based on the assessments on their respectively defined population sets. Adverse events (AEs) that emerged during treatment were monitored throughout the study until 30 days after discontinuation of the single agent and 150 days after discontinuation of the combination. Adverse events were coded using the Medical Dictionary for Regulatory Activities (MedDRA) and assessed as per Common Terminology Criteria for Adverse Events (CTCAE) version 4.03. The clinical laboratory assessments were collected no later than 30 days after the last date of study treatment administration. The grades were classified as low/normal/high when not defined by CTCAE v4.03. Other assessments of vital signs, physical examinations, and electrocardiogram (ECG) parameters were also performed.

Antitumor activity was locally assessed as per RECIST v1.1 and immune-related response criteria (irRC) (only for combination arm) and evaluated using the following endpoints: best overall response, overall response rate (ORR), disease control rate (DCR), time to tumor progression, and overall survival (OS). Estimation of best overall response (BOR) was based on assessments collected no later than 30 days after the last study treatment administration date for RECIST v1.1 and 150 days for irRC.

Pharmacokinetic endpoints included plasma concentrations and PK parameters of FGF401 and spartalizumab. Pharmacokinetic parameters were determined with noncompartmental method(s) using Phoenix WinNonlin version 6.4 or above (Pharsight Corporation, Mountain View, Califormia, USA). Immunogenicity of spartalizumab was assessed through presence and/or concentration of anti-drug (spartalizumab) antibodies (ADA). Food effect on FGF401 exposure was evaluated through plasma concentration of FGF401 and relevant PK parameters when dosing under fed condition. Safety of the food effect was assessed through incidence and severity of AEs and serious AEs (SAEs), including changes in laboratory values, vital signs, and ECGs when dosing under fed condition.

Additionally, exploratory biomarker endpoints to assess FGF19-FGFR4 signaling inhibition by FGF401 included temporal profiling of C4, FGF19, total bile acid and total cholesterol levels in blood, and *CYP7A1* and *DUSP6* transcript levels in tumor biopsies. An immunohistochemistry (IHC) assay to detect FGF19 was used retrospectively on remnant samples and the association of FGF19 expression levels with best change in sum of diameters (SOD) for RECIST v1.1 in the single-agent was evaluated.

### Statistical analysis

Study data were summarized with respect to demographic and baseline characteristics, efficacy, safety and all relevant pharmacokinetic and pharmacodynamic observations and measurements. Categorical data were presented as contingency tables (frequencies and percentages). For continuous data, summary statistics of mean (standard deviation) and median (minimum, maximum) were presented. The study data were analyzed and reported based on patient data from phase 1 and phase 2, up to the time when all patients had potentially completed at least 6 cycles of treatment or discontinued the study. The full analysis set and safety set comprised all patients who had received at least 1 dose of study medication.

For the single-agent phase 1 primary outcome, an extended Bayesian hierarchical logistical regression model (BHLRM) was applied to estimate the relationship between dose and stratum (fasted vs fed) specific probability of a patient experiencing a DLT. The results are summarized in terms of the probabilities that the true rate of DLT for each condition at each dose level will lie within each of the following intervals: (i) underdosing [0, 0.16); (ii) targeted toxicity [0.16, 0.33); and (iii) excessive toxicity [0.33, 1.00]. Under EWOC, any dose including MTD/RP2D, for which the DLT rate had more than a 25% risk of being excessively toxic, ie, P (DLT) ≥ 0.33 was not considered for the next dose cohort. The final reports based on DDS at the time of database lock included the following: (i) a plot of posterior interval probabilities; (ii) summary of the DLTs with onset during the evaluation period (phase 1 part only) by primary system organ class and preferred term; (iii) listing of inferential results from BHLRM.

The primary and secondary efficacy outcomes were analyzed in the full analysis set according to the assigned treatment stratified by study group, which we defined as a subgroup (fast/fed, group 1/2/3) of patients who were administered with similar dose levels on a given treatment arm and phase. The results have been presented as per RECIST v1.1. The number of patients having BOR was provided along with ORR and DCR with 95% exact binomial CIs (Clopper-Pearson). Waterfall plots for the phase 1 parts depicted the best percentage change from baseline in sum of longest diameters (SOD). The graphically presented Kaplan-Meier (KM) plots were used to estimate the TTP curve with one-sided 90% CI (Greenwood method) at 1.5 months, 3 months, and 6 months for the single-agent arm dosed at MTD/RP2D; the combination arm did not qualify for this analysis since it had fewer than 10 patients enrolled at RP2D. The estimate at a given time point was the estimated probability that a patient would remain event free up to that time point. A positive trend regarding the activity of FGF401 in groups 1 and 2 was concluded if the observed lower limit of their respective 90% CI was no less than their expected median TTPs of 1.5 months and 2.2 months estimated in the absence of FGF401. KM plots were also used to estimate OS rate with one-sided 95% CI at 3 months, 5 months, 7 months, and 9 months for single agent phase 1 overall and phase 2: groups 1, 2; for single agent phase 2 – group 3, OS, and progression free survival (PFS) were calculated regardless of RP2D but for overall tumor type.

Safety analyses conducted in the safety set constituted summaries for AEs occurring during on-treatment period with number and percentage of patients having at least 1 AE. Summaries presented overview of AEs and deaths (number and percentage of patients who died with any AE, SAE, dose reductions/interruptions, AE leading to discontinuation), SAEs, AEs related to study treatment, AEs leading to treatment discontinuation, dose interruption/adjustment or fatal outcome. Additionally, in combination part, summaries were produced using all treatment-related AEs starting or worsening during the on-treatment or extended safety follow-up periods (within 150 days after the date of last administration of study treatment).

The PK analyses and summary statistics were based on pharmacokinetic analysis set. Individual as well as mean concentration-time profiles were constructed using concentration data for spartalizumab and FGF401. Descriptive statistics for PK parameters included mean, standard deviation, coefficient of variation (CV%) mean, geometric mean, median, minimum, and maximum, with only the latter 3 for time to reach maximum concentration (T_max_) and additional 90% CI for accumulation ratio (R_acc_). Zero concentrations were not included in the geometric mean calculations. Missing concentrations or PK parameter values were not imputed.

Exploratory biomarker analyses were conducted using full analysis set to assess efficacy and FGF19 signaling inhibition associated with FGF401. The best tumor percentage change from baseline as per RECIST v1.1 were investigated by FGF19 status (positive/negative) as determined through RT-qPCR and IHC assay. An evaluable RT-qPCR sample was considered FGF19+ if mean threshold cycle for FGF19 was ≤35, and FGF19- otherwise. Among the patients with evaluable IHC assay samples, those who had % cellular positivity > 0 were considered FGF19 + .

### Biomarker assays methods

#### FGF19, FGFR4, KLB RT-qPCR

RNA was extracted from FFPE tissue biopsies using the Maxwell CSC RNA FFPE Kit (Promega cat #AS1360). Extracted RNA was reverse-transcribed into cDNA by means of High Capacity cDNA RT Kit (Thermo Fisher Scientific cat. #4368813) utilizing MultiScribe™ Reverse Transcriptase, according to manufacturer protocols. Gene expression of select genes of interest was evaluated by means of TaqMan® gene expression assays (Thermo Fisher Scientific cat. # 4364338) on a Applied Biosystems ViiA™ 7 detection platform. The genes of interest in the FGF401 expression panel included: FGF19: Hs00192780_m1, FGFR4: Hs01107438_m1, KLB: Hs01573147_m1. HUWE: Hs00948075_m1 was utilized as the endogenous reference control gene. The sample was considered evaluable if cycle threshold for control gene (HUWE1) was ≤35.

#### FGF19 IHC

An IHC assay using the Spring Bioscience anti-FGF19 antibody (clone SP268) was developed on HCC using Ventana OptiView detection on the Ventana BenchMark ULTRA staining platform (Ventana Medical Systems, Inc., USA). As negative control, specimens were incubated with an IgG rabbit monoclonal antibody under the same conditions. Anti-FGF19 antibody was detected using the OptiView™ detection kit (VMSI, cat. # 760–700). The OptiView Amplification Kit (VMSI, cat. #860–099) was utilized to improve sensitivity and enhance the intensity of the stain.

#### Blood-based biomarkers

To determine the circulating levels of the blood-based biomarkers FGF19, C4: 7α-Hydroxy-4-cholesten-3-one and total bile acids, serum samples were utilized and the various assays were performed at qualified vendors. FGF19 was quantified using a capture-ELISA assay from R&D System, Cat. nr. DF1900, with a LLOQ of 27.2 pg/mL and ULOQ of 1096.3 pg/mL. C4: 7α-Hydroxy-4-cholesten-3-one was determined using an LC-MS/MS method. The concentration of total bile acids were measured using the colorimetric diazyme’s enzymatic total bile acids assay.

#### RNAseq

For RNAseq based studies, extracted total RNA was depleted from ribosomal RNA using RNAseH. The rRNA-depleted sample was then prepared for sequencing using the TruSeq RNA v.2 Library Preparation kit (Illumina). After pooling the captured library using unique adapter index sequences and applying the pool to a sequencing flow cell, they were sequenced using Illumina v.4 chemistry and paired-end 100-bp reads. Sequence data were aligned to the reference human genome (build hg19) using STAR v.2.4.0e [27]. Mapped reads were then used to quantify transcripts with HTSeq v.0.6.1p1 [28] and RefSeq GRCh38 v.82 gene annotation. Gene expression data were normalized using the trimmed mean of M-value normalization as implemented in the edgeR – Bioconductor package v.3.20.9 [29].

## Results

### Study participants

In phase 1 of the study, 74 patients received FGF401 as a single agent, under fasting (*n* = 50) or fed conditions (*n* = 24), of whom 61 had HCC and 13 had other solid tumors (adenocarcinoma [*n* = 7 (pancreas:3, bile duct:2, stomach:1, hepatic neuroendocrine tumor:1)], cholangiocarcinoma [*n* = 4 (bile duct:3, liver:1)], and squamous cell carcinoma [*n* = 2 (left recto vaginal wall:1, thymic:1)]). In phase 2, 30 patients with HCC in group 1 and 36 in group 2 received FGF401. In group 3, 20 patients with other solid tumors (adenocarcinoma [*n* = 12 (pancreas:7, bile duct:2, kidney:1, prostate:1, urachus:1)], cholangiocarcinoma [*n* = 5 (bile duct:4, liver:1)], others [*n* = 3 (liver:1, thyroid:1, adrenal:1)]) were enrolled.

Of 139 patients with HCC, all BCLC stage C and a median (min-max) of 2 (0–25) liver nodules, half (~ 50%, *n* = 66) of them had liver nodule ≥5 cm; 77% (*n* = 107) presented with tumor lymph node metastasis stage IVA or IVB and largely moderately or well differentiated histology.

All 160 patients in FGF401 single agent discontinued the treatment: progressive disease (PD) in 130 (81%) being the most common reason, followed by AEs in 18 patients (11%) (Supplementary Table 1/Fig. 1). Patients had a median age of 62 years (range, 21–85 years), with males representing 74% of the patients. The patients were mainly Asians (46%) or Caucasians (39%) with an ECOG performance status of “0” in 44% of patients. Of these, 124 patients (77.5%) were previously treated with protein kinase inhibitors and 5 patients (3.1%) were treated with immune-checkpoint inhibitors.

Among the 12 patients who were treated with the combination of FGF401 with spartalizumab and dosed under fasting conditions, 9 (75%) discontinued due to PD. The median age of patients was 65 years (range, 44–78 years), with the majority being Asians (8 [67%]) and 42% having ECOG status “1”. All the 12 patients were treated previously with protein kinase inhibitors and only 1 (8.3%) with immune checkpoint inhibitor (Table 1).

**Table 1:** Demographics and baseline disease characteristics of patients receiving FGF401 as single agent or with spartalizumab

Demographics	Phase 1 single agent						Phase 2 single agent			All patients ***N*** = 160	Combination		
	50 mg; *N* = 11 Fasted	80 mg; *N* = 6 Fasted	80 mg; *N* = 5 Fed	120 mg; *N* = 26 Fasted	120 mg; *N* = 19 Fed	150 mg; *N* = 7 Fasted	Group 1 *N* = 30	Group 2 *N* = 36	Group 3 *N* = 20		FGF401 80 mg + spartalizumab 300 mg *N* = 6	FGF401 120 mg + spartalizumab 300 mg *N* = 6	All Patients *N* = 12
Age, median, years	64.0 (38–76)	59.0 (40–66)	61.0 (24–75)	61.5 (36–80)	60.0 (23–85)	63.0 (44–79)	60.5 (37–81)	65.5 (41–81)	65.0 (21–79)	62.0 (21–85)	66.0 (53–78)	65.0 (44–73)	65.0 (44–78)
Sex, male, n (%)	7 (63.6)	5 (83.3)	4 (80.0)	23 (88.5)	13 (68.4)	7 (100)	22 (73.3)	29 (80.6)	8 (40.0)	118 (73.8)	5 (83.3)	2 (33.3)	7 (58.3)
Race, n (%)													
Asian	9 (81.8)	2 (33.3)	2 (40.0)	14 (53.8)	10 (52.6)	4 (57.1)	30 (100)	0	2 (10.0)	73 (45.6)	3 (50.0)	5 (83.3)	8 (66.7)
Caucasian	2 (18.2)	4 (66.7)	3 (60.0)	12 (46.2)	8 (42.1)	1 (14.3)	0	21 (58.3)	11 (55.0)	62 (38.8)	3 (50.0)	1 (16.7)	4 (33.3)
Others	0	0	0	0	1 (5.3)	2 (28.6)	0	15 (41.7)	7 (35.0)	25 (15.6)	0	0	0
BMI, median, (kg/m^2^)	19.9 (19–27)	25.2 (24–34)	22.9 (20–29)	24.6 (16–33)	23.2 (19–29)	21.5 (18–35)	22.7 (17–31)	25.8 (14–33)	24.3 (17–33)	24.2 (14–35)	26.2 (20–36)	22.4 (19–33)	25.3 (19–36)
ECOG performance status, n (%)													
0	3 (27.3)	4 (66.7)	1 (20.0)	12 (46.2)	8 (42.1)	2 (28.6)	10 (33.3)	21 (58.3)	10 (50.0)	71 (44.4)	5 (83.3)	2 (33.3)	7 (58.3)
1	8 (72.7)	2 (33.3)	4 (80.0)	14 (53.8)	11 (57.9)	5 (71.4)	20 (66.7)	15 (41.7)	10 (50.0)	89 (55.6)	1 (16.7)	4 (66.7)	5 (41.7)
Previous therapy*													
Surgery	9 (81.8)	2 (33.3)	4 (80.0)	16 (61.5)	12 (63.2)	5 (71.4)	15 (50.0)	24 (66.7)	13 (65.0)	100 (62.5)	5 (83.3)	4 (66.7)	9 (75.0)
Radiotherapy	4 (36.4)	1 (16.7)	3 (60.0)	9 (34.6)	6 (31.6)	3 (42.9)	15 (50.0)	7 (19.4)	7 (35.0)	55 (34.4)	2 (33.3)	1 (16.7)	3 (25.0)
Medication	10 (90.9)	5 (83.3)	5 (100)	25 (96.2)	17 (89.5)	6 (85.7)	30 (100)	36 (100)	20 (100)	154 (96.3)	6 (100)	6 (100)	12 (100)
*Protein kinase inhibitors (axitinib, imatinib mesilate, lenvatinib, sorafenib, sorafenib tosylate, sunitinib)*	7 (63.6)	4 (66.7)	5 (100)	20 (76.9)	14 (73.7)	5 (71.4)	30 (100)	36 (100)	3 (15.0)	124 (77.5)	6 (100)	6 (100)	12 (100)
*Monoclonal antibodies (nivolumab, pembrolizumab, ramucirumab, panitumumab)*	0	0	0	0	3 (15.8)	0	0	0	2 (10.0)	5 (3.1)	0	1 (16.7)	1 (8.3)

*BMI* Body mass index, *ECOG* Eastern Cooperative Oncology Group

*A patient may have multiple settings

### MTD/RP2D identification

In phase 1 dose-escalation of single-agent FGF401, 74 patients received FGF401 once daily at 4 dose levels (50 mg, 80 mg, 120 mg, and 150 mg) under fasted conditions and at 2 dose levels (80 mg and 120 mg) under fed conditions. From the 70 patients included in DDS, 6 patients experienced DLT: 1 grade 3 alanine aminotransferase (ALT) increase (*n* = 1, 50 mg, fasted), 1 grade 3 aspartate aminotransferase (AST) increase (*n* = 1, 120 mg, fasted), 1 grade 2 and 1 grade 3 blood bilirubin increase (*n* = 1, 120 mg, fed); 2 grade 3 AST increase (*n* = 2, 150 mg, fasted) and 1 grade 3 ALT and 1 grade 3 AST (*n* = 1, 150 mg, fasted). MTD was not reached and RP2D for FGF401 as a single agent in fasting or fed conditions was determined as 120 mg qd based on overall clinical safety/tolerability, efficacy, and PK/PD results.

In the combination part, spartalizumab was administered at a fixed dose of 300 mg intravenously every 3 weeks, while FGF401 was administered orally at 80 mg qd in 6 patients and 120 mg qd in the other 6 patients. No DLTs were reported and MTD was not evaluated while RP2D for the combination part was determined as 120 mg FGF401 + 300 mg spartalizumab.

### Safety and tolerability

In the single-agent FGF401 arm, duration of exposure varied by treatment dose and group, with a median of 11 weeks (range, 0.1–135.3 weeks). Fifty patients (31.3%) had an exposure of ≤6 weeks, while 11 patients (6.9%) had an exposure of > 52 weeks (phase 1 [*n* = 4]: 80 mg fed [*n* = 1], 120 mg fasted [*n* = 2], 150 mg fasted [*n* = 1]; phase 2 [*n* = 7]: group 1 [*n* = 1], group 2 [*n* = 5], group 3 [*n* = 1]). A median of 1 dose interruption was observed in 97 patients (60.6%), primarily due to AEs (52.5%) and dose reduction in 34 patients (21.3%) also primarily due to AEs (18.1%). Eighteen patients (11.3%) had an AE leading to discontinuation of FGF401 treatment. Most frequently occurring AEs irrespective of study treatment relationship which led to FGF401 discontinuation were: ALT increased in 6 patients (3.8%), blood bilirubin increased in 4 patients (2.5%), and AST increased in 3 patients (1.9%).

In FGF401 single-agent arm, 116 of 160 patients (72.5%) had a grade 3 or 4 AE (Table 2). The most frequent AEs occurring in ≥30% of patients irrespective of the relationship with study treatment were diarrhea (118 [73.8%]), elevated AST (76 [47.5%]), and increased ALT (70 [43.8%]). Grade 3 or 4 AEs suspected to be related to study treatment were reported in 51 patients (31.9%); most frequent were AST increased 30 (18.8%), ALT elevated 24 (15.0%), and diarrhea 8 (5.0%). The safety profile appeared comparable for FGF401 administered under both fed and fasting conditions (Table 3). Twenty patients (12.5%) died on treatment in the FGF401 single-agent arm: 17 patients (10.6%) because of existing cancer (HCC [*n* = 10], cholangiocarcinoma [*n* = 4], adenocarcinoma pancreas [*n* = 2], neoplasm of thymus [*n* = 1]) and 3 patients because of other reasons (gastric bleeding, multiple organ dysfunction syndrome, unknown cause of death).

**Table 2:** Overall AEs in FGF401 single agent and FGF401 + spartalizumab combination arm

Category	FGF401 single agent All patients ***N*** = 160		FGF401 + spartalizumab All patients ***N*** = 12	
	All grades n (%)	Grade 3 or 4 n (%)	All grades n (%)	Grade 3 or 4 n (%)
AEs	160 (100)	116 (72.5)	12 (100)	6 (50.0)
Treatment related	148 (92.5)	51 (31.9)	11 (91.7)	4 (33.3)
SAEs	70 (43.8)	55 (34.4)	2 (16.7)	2 (16.7)
Treatment related	8 (5.0)	7 (4.4)	2 (16.7)	1 (8.3)
Fatal SAEs	3 (1.9)	3 (1.9)	0	0
Treatment related	0	0	0	0
AEs leading to FGF401 discontinuation	18 (11.3)	13 (8.1)	2 (16.7)	1 (8.3)
Treatment related	12 (7.5)	8 (5.0)	1 (8.3)	0
AEs leading to FGF401 dose adjustment/interruption	80 (50.0)	58 (36.3)	4 (33.3)	3 (25.0)
Treatment related	47 (29.4)	34 (21.3)	2 (16.7)	2 (16.7)

*AE* Adverse event, *SAE* Serious adverse event

**Table 3:** Adverse events in FGF401 single-agent arm regardless of study treatment relationship by preferred term*

Preferred term	Phase 1 part												Phase II part						All patients	
	50 mg		80 mg				120 mg				150 mg		Group 1		Group 2		Group 3			
	Fasted		Fasted		Fed		Fasted		Fed		Fasted									
	***N*** = 11		***N*** = 6		***N*** = 5		***N*** = 26		***N*** = 19		***N*** = 7		***N*** = 30		***N*** = 36		***N*** = 20		***N*** = 160	
	All grades	Grade 3/4	All grades	Grade 3/4	All grades	Grade 3/4	All grades	Grade 3/4	All grades	Grade 3/4	All grades	Grade 3/4	All grades	Grade 3/4	All grades	Grade 3/4	All grades	Grade 3/4	All grades	Grade 3/4
	n (%)	n (%)	n (%)	n (%)	n (%)	n (%)	n (%)	n (%)	n (%)	n (%)	n (%)	n (%)	n (%)	n (%)	n (%)	n (%)	n (%)	n (%)	n (%)	n (%)
Diarrhea	8 (72.7)	2 (18.2)	4 (66.7)	0	4 (80.0)	0	18 (69.2)	0	14 (73.7)	0	7 (100)	0	23 (76.7)	2 (6.7)	23 (63.9)	3 (8.3)	17 (85.0)	1 (5.0)	118 (73.8)	8 (5.0)
Nausea	1 (9.1)	0	1 (16.7)	0	0	0	5 (19.2)	0	6 (31.6)	0	1 (14.3)	0	7 (23.3)	0	6 (16.7)	0	11 (55.0)	0	38 (23.8)	0
Abdominal pain	0	0	1 (16.7)	0	3 (60.0)	1 (20.0)	3 (11.5)	0	6 (31.6)	1 (5.3)	0	0	5 (16.7)	0	8 (22.2)	1 (2.8)	9 (45.0)	3 (15.0)	35 (21.9)	6 (3.8)
Vomiting	1 (9.1)	0	1 (16.7)	0	1 (20.0)	0	4 (15.4)	0	6 (31.6)	0	1 (14.3)	0	5 (16.7)	0	4 (11.1)	0	9 (45.0)	1 (5.0)	32 (20.0)	1 (0.6)
Constipation	1 (9.1)	0	0	0	1 (20.0)	0	4 (15.4)	0	1 (5.3)	0	0	0	5 (16.7)	0	5 (13.9)	0	6 (30.0)	0	23 (14.4)	0
Ascites	1 (9.1)	1 (9.1)	1 (16.7)	1 (16.7)	2 (40.0)	1 (20.0)	6 (23.1)	4 (15.4)	1 (5.3)	1 (5.3)	2 (28.6)	0	4 (13.3)	1 (3.3)	3 (8.3)	1 (2.8)	2 (10.0)	1 (5.0)	22 (13.8)	11 (6.9)
Abdominal distension	1 (9.1)	0	0	0	1 (20.0)	1 (20.0)	3 (11.5)	0	0	0	3 (42.9)	0	4 (13.3)	0	1 (2.8)	0	1 (5.0)	0	14 (8.8)	1 (0.6)
Pyrexia	3 (27.3)	0	0	0	2 (40.0)	0	6 (23.1)	0	1 (5.3)	0	1 (14.3)	0	9 (30.0)	0	4 (11.1)	0	6 (30.0)	0	32 (20.0)	0
Fatigue	2 (18.2)	0	1 (16.7)	0	0	0	6 (23.1)	1 (3.8)	5 (26.3)	1 (5.3)	0	0	2 (6.7)	0	8 (22.2)	0	7 (35.0)	0	31 (19.4)	2 (1.3)
Edema peripheral	0	0	1 (16.7)	0	0	0	10 (38.5)	0	0	0	0	0	8 (26.7)	0	9 (25.0)	0	3 (15.0)	0	31 (19.4)	0
Asthenia	2 (18.2)	0	2 (33.3)	1 (16.7)	0	0	4 (15.4)	0	3 (15.8)	1 (5.3)	2 (28.6)	0	4 (13.3)	1 (3.3)	6 (16.7)	2 (5.6)	4 (20.0)	2 (10.0)	27 (16.9)	7 (4.4)
Aspartate aminotransferase increased	4 (36.4)	2 (18.2)	3 (50.0)	0	2 (40.0)	1 (20.0)	16 (61.5)	9 (34.6)	14 (73.7)	3 (15.8)	7 (100)	5 (71.4)	14 (46.7)	8 (26.7)	11 (30.6)	5 (13.9)	5 (25.0)	4 (20.0)	76 (47.5)	37 (23.1)
Alanine aminotransferase increased	3 (27.3)	2 (18.2)	3 (50.0)	0	2 (40.0)	2 (40.0)	14 (53.8)	4 (15.4)	11 (57.9)	3 (15.8)	7 (100)	3 (42.9)	13 (43.3)	5 (16.7)	11 (30.6)	6 (16.7)	6 (30.0)	4 (20.0)	70 (43.8)	29 (18.1)
Blood bilirubin increased	4 (36.4)	1 (9.1)	3 (50.0)	0	1 (20.0)	0	11 (42.3)	3 (11.5)	2 (10.5)	2 (10.5)	0	0	6 (20.0)	3 (10.0)	3 (8.3)	2 (5.6)	1 (5.0)	1 (5.0)	31 (19.4)	12 (7.5)
Decreased appetite	3 (27.3)	0	1 (16.7)	0	0	0	10 (38.5)	0	2 (10.5)	0	4 (57.1)	0	6 (20.0)	0	9 (25.0)	0	12 (60.0)	0	47 (29.4)	0
Pruritus	2 (18.2)	0	2 (33.3)	0	1 (20.0)	0	4 (15.4)	0	5 (26.3)	0	1 (14.3)	0	5 (16.7)	0	8 (22.2)	1 (2.8)	0	0	28 (17.5)	1 (0.6)

- A patient with multiple occurrences of an AE under 1 treatment is counted only once in the AE category for that treatment

*In ≥30% of patients, safety set

For FGF401 + spartalizumab, median duration of exposure to study treatment was 19.6 weeks (range, 6.0–57.0 weeks). Dose interruption of FGF401 was observed in 7 patients (58.3%), primarily because of AEs in 4 patients (33.3%) and dose interruption of spartalizumab in 2 patients (16.7%), both due to AEs. All 12 patients had at least 1 AE irrespective of the relationship to study treatment, of whom 6 patients (50.0%) had a grade 3 or 4 AE (Table 2). The most frequent AEs irrespective of the relationship with study treatment were diarrhea in 7 (58.3%), AST increased in 6 (50.0%), hyperphosphatemia in 5 (41.7%), ALT increased, pyrexia and anemia in 4 each (33.3%) of the patients enrolled in combination arm. Eleven of the 12 patients (91.7%) had an AE suspected to be related to study treatment, which was a grade 3 or 4 AE in 4 patients. Two patients had a grade 3 or 4–related event of diarrhea, while a grade 3 or 4–related event of AST increased, hyperglycemia, platelet count decreased, and anemia occurred in 1 patient each. There was no on-treatment death in the FGF401 + spartalizumab arm. Two patients treated with 80 mg FGF401 + 300 mg spartalizumab and 2 patients treated with 120 mg FGF401 + 300 mg spartalizumab died more than 30 days after the last treatment due to PD.

### PK and immunogenicity results

**Fig. 2:**
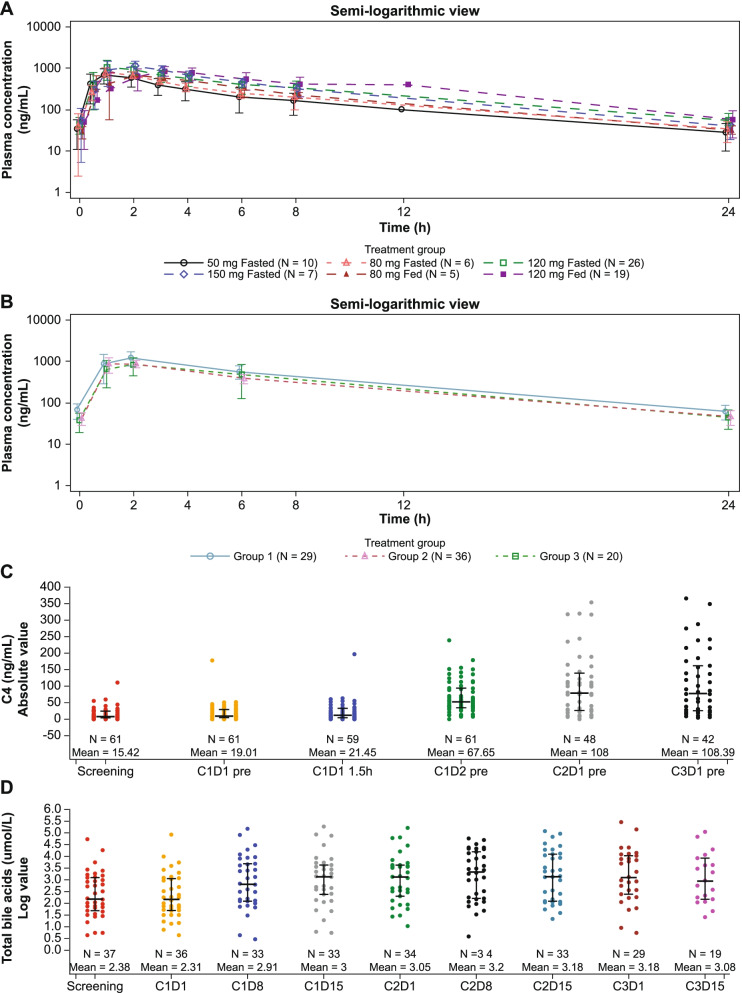
Pharmacokinetics of FGF401 and blood pharmacodynamics in patients treated with FGF401 as a single agent. **A** Plasma concentrations of FGF401 over time are shown in a semi-log view for phase 1 cycle 1 day 8 and **B** phase 2 cycle 2 day 1. **C** Bile acid precursor C4 and **D** total bile acid levels increased after treatment reflecting de-repression of bile acids synthesis as a consequence of FGFR4 pathway inhibition. C4 and total bile acid are shown at different days of cycle for the patients with HCC in the 120 mg fasted dose group. C4: 7α-hydroxy-4-cholesten-3-one bile acid

FGF401 was rapidly absorbed upon administration, with median T_max_ varying in phase 1 from 1.00 to 2.98 hours on cycle 1 day 1 (C1D1), 1.00 to 3.01 hours on cycle 1 day 8 (C1D8, Fig. 2A and Supplementary Table 2) and 1.01 to 3.02 hours on cycle 2 day 1 (C2D1). The terminal half-life (T_1/2_) of FGF401 ranged from 4.91 to 6.57 hours over all treatments and during all periods. Both T_max_ and T_1/2_ appeared independent of dose and unchanged after repeated dosing. Based on T_1/2_, steady state was considered to have been reached before C1D8 after repeated dosing. The observed area under the curve (AUC) and maximum plasma concentration (C_max_) on C1D8 and C2D1 were comparable to those on C1D1, with R_acc_ around 1. This indicated that no drug accumulation of FGF401 occurred following repeated dosing, which agrees with the short-to-moderate T_1/2_.

Plasma drug exposures (AUC and C_max_) increased with an ascending dose. A dose-proportionality test suggested that exposures increased slightly under dose proportion from 50 to 150 mg (fasted and fasted-fed combined), with a smaller sample size at 50 mg and 150 mg dose levels than at 80 mg and 120 mg doses. Typically, at the 120 mg fasted dose, the geometric mean (CV%) of C_max_ and drug exposure within a dosing interval (AUC_tau_) at steady state (C1D8) were 1120 ng/mL (36.5%) and 6970 h*ng/mL (38.7%), respectively. The overall interpatient variability of drug exposure was moderate for FGF401. No PK parameter was calculated for the phase 2 part because of sparse sampling, however, the plasma concentrations obtained from phase 2 patients were very close to those obtained from phase 1 patients with 120 mg qd fasted dose regimen (Fig. 2B).

Food effect was tested at 80 mg and 120 mg dose of FGF401 in the dose-escalation part. Plasma drug exposures were comparable between fed and fasted conditions at both concentrations, and there was no effect on half-life, though slightly delayed T_max_ occurred when FGF401 was taken with low fat meals (Fig. 2A). The AEs regardless of relationship with study drug were comparable between fed and fasted conditions (Table 3). Though the assessment was not designed for stringent statistical tests, it was concluded that there was no food effect on drug exposure, safety, and tolerability when taking FGF401 with a low-fat meal.

The PK profiles of FGF401 in combination with spartalizumab were similar to those of single-agent FGF401 (Supplementary Fig. 1). For spartalizumab, geo-mean AUC_last_ was 795 d*μg/mL in combination with 80 mg FGF401 and 978 d*μg/mL in combination with 120 mg FGF401. Geo-mean C_max_ was 74.7 μg/mL and 87.7 μg/mL, respectively (Supplementary Table 3). Though the data were limited for the combination arm, it appeared that the spartalizumab exposures in combination with FGF401 might be comparable with the spartalizumab exposures from similar studies with the same 300 mg q3w dose regimen [25, 30]. There seemed no drug-drug interaction between FGF401 and spartalizumab to alter the PK profiles of each other in the combination arm.

One of 12 patients in the FGF401 and spartalizumab combination part (8.3%) was positive for treatment-induced ADA for spartalizumab in 2 IG samples collected at C2D1 and C3D1 predose, respectively. The ADA titers were low to moderate. The potential ADA did not appear to influence the PK profiles of the test compounds in this patient.

### Efficacy

**Fig. 3:**
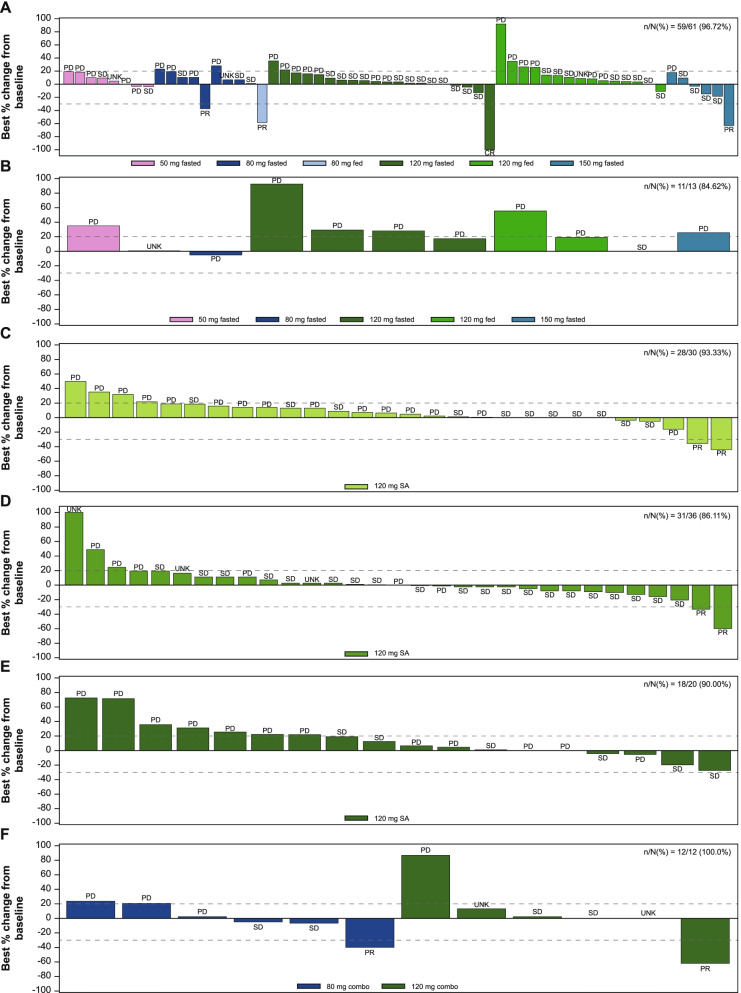
Response to FGF401. Waterfall plot for best percentage change from baseline in sum of the longest diameters based on local radiology review per RECIST v1.1 in **A** single agent phase I patients with HCC, **B** single agent Phase I patients with other solid tumors, **C** single agent Phase II group 1 patients with HCC [Asian countries], **D** single agent Phase II group 2 patients with HCC [non-Asian countries], **E** single agent Phase II group 3 patients with other solid tumors, **F** combination phase I patients with HCC. PD, progressive disease; PR, partial response; SD, stable disease; UNK, unknown

Of 59 evaluable patients with HCC in phase 1 of single agent FGF401, complete response (CR) was achieved in 1 patient (120 mg, fasted) and partial response (PR) in 3 (80 mg fasted, 80 mg fed, 150 mg fasted, each) (Fig. 3A). There were no responses in patients with other tumor types (Fig. 3B). In phase 2 group 1 (*n* = 28), 2 patients had PR, 11 patients had stable disease (SD) (Fig. 3C); in group 2 (*n* = 31), 2 patients had a PR and 20 had SD (Fig. 3D), and in group 3 (*n* = 18), 6 had SD (Fig. 3E).

The median TTP for patients treated with 120 mg FGF401 in phase 1 (under fed and fasted conditions combined; *N* = 45) was 2.63 months and median OS of 5.72 months (n/*N* = 38/45) (Supplementary Fig. 2F). In phase 2 FGF401 single agent, the probability estimates of remaining event free (90% CI) at 9 months was maximum for group 2 at 61.5 (49.8, 100) followed by group 1 (30.4 [19.1, 100] and group 3 (24.7 [12.6, 100]).

In each cohort of the combination arm (80 mg FGF401 + 300 mg spartalizumab and 120 mg FGF401 + 300 mg spartalizumab), there was 1 patient with a PR and 2 patients with SD; with DCR of 50.0% (6 of 12; 95% CI: 11.8–88.2) (Fig. 3F). In 120 mg FGF401 + 300 mg spartalizumab combination arm, 2 of the 6 patients (33.3%) had an event. Owing to the small number of patients, OS was not summarized for the combination arm.

### Biomarker results

In the FGF401 single-agent arm, patients showed varying levels of FGF19 transcript in the biopsy obtained for molecular prescreening, with no clear association with response (Supplementary Fig. 2A, B). Among patients with HCC in phase 1/2 of single agent, for which FGF19 IHC analysis was performed, 27 were FGF19 positive and 33 were FGF19 negative with a trend for better response among the FGF19 IHC-positive patients (Supplementary Fig. 2C, D). The comparison of the IHC data to the RT-qPCR data for FGF19 revealed a disagreement between the 2 assays for a few samples. In particular, 7 FGF19 IHC-positive samples were negative by RT-qPCR and 2 FGF19 IHC-negative samples were positive by RT-qPCR (Supplementary Fig. 2E). Some of the IHC results showed very focal (less than 1% of the tumor), but strong protein expression. Based on this observation, we hypothesize that a reason for this difference between the RNA and IHC positivity could be due to the fact that this focal RNA expression within a tumor lysate did not meet detection threshold and that the visual context of IHC testing was needed in these situations.

In order to try to identify more robust biomarkers for response, the RNA from pretreatment and on-treatment tumor biopsies obtained at C1D15 was sequenced and genome-wide transcriptome analysis was performed. Correlation analyses utilizing baseline gene expression data or the fold change between on-treatment and baseline paired biopsies did not reveal signals that enriched for response as measured by best percent tumor change or PFS. Similarly, a differential gene expression analysis comparing the group of FGF401 responders versus the group of non-responders failed to elicit gene signatures that were significantly associated with outcome.

FGFR4/KLB pathway activation by FGF19 promotes bile acid production from cholesterol. Accordingly, in phase 1 and phase 2 of the FGF401 single-agent arm, treatment-induced elevation of C4 and total bile acid was observed in most patients across the FGF401 dose levels (Fig. 2C, D). In addition, an increase in circulating FGF19, as a feedback–loop response to the elevated bile acids, and decrease in total cholesterol were detected (Supplementary Fig. 3A, B). Consistent with the FGF19/FGFR4 downstream pathway, tumor PD assessed by RNAseq revealed an upregulation of CYP7A1 transcript concomitant with downregulation of the MAPK target gene *DUSP6*, in most on-treatment biopsies obtained at C1D8 with respect to the matched pretreatment sample (Supplementary Fig. 3C, D). These results were indicative of FGFR4 pathway suppression being achieved at all doses tested. Dose-response analyses with the pharmacodynamic biomarkers did not identify any associations. Heatmap showing the expression levels for DUSP6 and CYP7A1 as well as signatures of immune infiltration [31] were summarized with GSVA [32] for both screening and on-treatment when available (Supplementary Fig. 4). We did not observe significant changes in immune infiltration after FGF401 treatment. Biopsy location and sources were not always identical within a sample pair, limiting the interpretability of these results. Moreover, high intratumor heterogeneity inherent to HCC may explain why in various instances, the suppression of the FGF19/FGFR4 pathway did not result in an objective tumor response [33].

## Discussion

In this first-in-human study, FGF401 demonstrated favorable pharmacokinetic characteristics with good oral absorption and moderate to short elimination half-life. Preclinically, FGF401 showed in vivo phospho-FGFR4 IC_90_ at 52.1 nM and anti-tumor efficacy was driven by a fraction of time above this IC_90_, or depending on the trough concentration levels [22]. This IC_90_ was equivalent to ~ 23 ng/mL in human plasma. For FGF401 with qd dosing, the drug concentrations at 24 h post-dose in Cycle 1 Day 8 (Fig. 2A) or Cycle 2 Day 1 (Fig. 2B) could be considered as the trough concentration at the steady state, the mean values of which are all approximately above this IC_90_ even at the 50 mg qd as shown in Fig. 2A. This finding indicates the favorable PK profiles of FGF401 at current clinical dose levels supported the potential linkage to its clinical PD and efficacy.

There was no food effect on PK, safety, and tolerability when dosing FGF401 with low-fat meals. FGF401 showed modest safety and tolerability; diarrhea and transaminases elevations were the most common treatment-related AEs observed under both fasted and fed conditions and were likely on-target effects of FGFR4 pathway inhibition. Consistent with this, diarrhea was treated with cholestyramine, a bile acid sequestrant. While the overall rate of diarrhea was high, the study treatment discontinuation from this AE was low. Discontinuation of study treatment and dose interruptions were more common from elevated transaminases.

Preliminary efficacy was observed with 4 patients reporting PR at RP2D in phase 2, all in patients with HCC. In addition, 1 CR and 3 PR were reported in phase 1 in patients with HCC. Overall, FGF401 suppressed the FGFR4 pathway at all treatment doses as assessed by modulation of C4, total bile acid, circulating FGF19 and cholesterol, without a clear dose response. Analysis of FGF19 expression and genome-wide transcriptome analysis in tumor samples indicated a trend for better responses in patients whose tumors were FGF19 IHC positive. A reliable biomarker to identify patients who are most likely to benefit was elusive from the data collected and analyzed for this study.

Targeting FGFR4 signaling has emerged as a potential treatment modality for effective, biomarker-driven treatment of HCC and other solid malignancies [19]. Recently, first-line therapies, such as lenvatinib, have shown inhibition of *FGF* pathways in patients with HCC [34], however, the specificity of the drug against the *FGF19*–*FGFR4* signaling pathway stays unclear [35]. FGF401 is one of the promising reversible FGFR4 inhibitor, which may have improved effects over irreversible inhibitors [36]. In a preclinical study, FGF401 was studied in combination with FGFR1–3 inhibitor, infigratinib, and found that HCC patients with high expression of FGFR2/3 or FGF19/FGFR4 might benefit from the combination if evaluated further in the clinical studies [37].

Other FGFR4 inhibitors in development and under evaluation are BLU9931/BLU554 [38, 39] and H3B-6527 [40–42]. In a first-in-human study with BLU-554 in patients with HCC, the ORR was 17% in FGF19-positive patients (median duration of response: 5.3 months [95% CI: 3.7-not reached]) and 0% in FGF19-negative patients, showing a correlation between tumor response and FGF19 expression. Treatment discontinuation due to AE was reported in 12% of patients comparable to 11.3% in our study. The most common treatment related AE (TRAE) in patients treated with BLU-554 qd were diarrhea (74%), nausea (42%), and vomiting (35%), related to enhanced bile-acid secretion. Grade ≥ 3 TRAEs occurred in 43% of the patients; the most common being transaminase elevation [19].

Interim analysis of Phase I study with H3B-6527 showed that, for HCC patients with > 2 prior lines of therapy treated on qd schedule, OS was 10.6 months, PFS 4.1 months, ORR 16.7% (all PR), and clinical benefit rate 45.8% (responders + durable SD > 17 weeks). Overall, for patients with HCC and intrahepatic cholangiocarcinoma, drug discontinuation due to AEs for qd dosing was 8.3% with most frequent treatment-emergent AE (TEAE) as diarrhea, fatigue, nausea. Grade 3 TEAE occurred in 12.5% of patients [41, 42].

The modest clinical activity in our study may be explained by the lack of a reliable biomarker, treatment inconsistency due to AEs, or co-dependence of HCC growth on other signaling pathways. Considering the immune-driven nature of HCC [43] and recent approval of immune checkpoint inhibitors for this cancer [6, 7, 12], we further evaluated the safety profile of FGF401 in combination with spartalizumab. Although the number of patients treated in the combination arm was limited, the safety, tolerability, and efficacy of the combination were similar to that observed with single-agent FGF401. Moreover, there was no drug-drug interaction between FGF401 and spartalizumab.

Addition of CLTA-4 inhibitor to PD-1 inhibitor is another promising treatment modality to harness the power of immune system to treat HCC [8, 44]. In an ongoing phase III study, PD-1 inhibitor, durvalumab is being evaluated as monotherapy and in combination with anti-CTLA-4, tremelimumab, in patients with unresectable HCC and has demonstrated a favorable benefit-risk profile [45]. A combination of the multikinase inhibitor, cabozantinib with PD-L1 inhibitor, atezolizumab significantly improved PFS when compared with sorafenib alone in the first-line treatment of advanced HCC (*p* = 0.0012) but there is no improvement in OS [44].

In summary, the study met its primary endpoint and the RP2D has been defined in this trial for FGF401 alone and in combination with spartalizumab. FGF401 demonstrated developable PK properties. Treatment with FGF401 was safe as a single agent and in combination with anti-PD1 therapy with common on-target AEs of transaminase elevation and diarrhea. The study has shown signals of efficacy as monotherapy and in combination with anti-PD1 therapy with evidence of FGFR4 pathway inhibition. Data show that further studies could help to better identify optimal drug combinations and predictive biomarkers.

## Conclusions

FGF401 demonstrated favorable pharmacokinetic characteristics with evidence of FGFR4 inhibition. FGF401 alone or in combination with spartalizumab had a manageable safety profile with AEs that are considered on-target effects of pathway inhibition. Clinical activity was observed in patients with HCC. No clear biomarker could be identified to robustly predict response and may be an area for further investigation.

## Supplementary Information

**Additional file 1.**

**Additional file 2.**

## Data Availability

Novartis will not provide access to patient-level data if there is a reasonable likelihood that individual patients could be reidentified. Phase 1 studies, by their nature, present a high risk of patient reidentification; therefore, patients’ individual results for phase 1 studies cannot be shared. In addition, clinical data, in some cases, have been collected patient to contractual or consent provisions that prohibit transfer to third parties. Such restrictions may preclude granting access under these provisions. Where co-development agreements or other legal restrictions prevent companies from sharing particular data, companies will work with qualified requestors to provide summary information where possible.

## Abbreviations

ADA: Anti-drug antibody
AE: Adverse event
ALT: Alanine aminotransferase
AST: Aspartate aminotransferase
AUC: Area under the curve
AUC_last_: Area under the plasma concentration-time curve from time zero to time of last measurable concentration
AUC_tau_: Area under the plasma concentration-time curve over dosing interval
BHLRM: Bayesian hierarchical logistical regression model
BOR: Best overall response
CI: Confidence interval
C_max_: Maximum plasma concentration
CR: Complete response
CT: Cycle threshold
CTCAE: Common Terminology Criteria for Adverse Events
CV%: Coefficient of variation
DCR: Disease control rate
DDS: Dose determining set
DLT: Dose-limiting toxicity
ECG: Electrocardiogram
ECOG: Eastern Cooperative Oncology Group
EWOC: Escalation with overdose control
FGF19: Fibroblast growth factor 19
FGFR4: Fibroblast growth factor receptor 4
HCC: Hepatocellular carcinoma
IHC: Immunohistochemistry
irRC: Immune-related response criteria
IV: Intravenous
KLB: B-Klotho
KM: Kaplan-Meier
MAPK: Mitogen-activated protein kinase
MedDRA: Medical Dictionary for Regulatory Activities
MKI: Multikinase inhibitors
MTD: Maximum tolerated dose
ORR: Overall response rate
OS: Overall survival
PD: Progressive disease
PD-1: Programmed cell death protein-1
PD-L1/L2: Programmed cell death ligand-1/2
PFS: Progression-free survival
PK: Pharmacokinetic
PR: Partial response
q3w: Once every three weeks
qd: Once daily
R_acc_: Accumulation ratio
RECIST: Response Evaluation Criteria in Solid Tumors
RP2D: Recommended phase 2 dose
RT-qPCR: Reverse transcription quantitative polymerase chain reaction
SA: Single agent
SAE: Serious adverse event
SD: Stable disease
T_1/2_: Elimination half-life
T_max_: Time to reach C_max_
TTP: Time to progression
