# Supplementary material for: A first-in-human phase 1/2 study of FGF401 and combination of FGF401 with spartalizumab in patients with hepatocellular carcinoma or biomarker-selected solid tumors
Source: J Exp Clin Cancer Res. 2022 Jun 2;41:189. doi: 10.1186/s13046-022-02383-5 (PMC9161616; doi:10.1186/s13046-022-02383-5)
Supplement: Supplementary file 1 — Additional file 1. [file 13046_2022_2383_MOESM1_ESM.docx]

Supplementary Table 1 *Patient disposition for FGF401 single agent and FGF401 + spartalizumab combination therapy arms*

| **Disposition** | **FGF401 single agent**  **N=160**  **n (%)** | **FGF401 +** **spartalizumab**  **N=12**  **n (%)** |
| --- | --- | --- |
| Treatment discontinued | 160 (100) | 12 (100) |
| **Primary reason for end of treatment** | | |
| Adverse event | 18 (11.3) | 2 (16.7) |
| Physician decision | 1 (0.6) | 0 |
| Progressive disease | 130 (81.3) | 9 (75.0) |
| Patient/guardian decision | 9 (5.6) | 1 (8.3) |
| Death | 2 (1.3) | 0 |

Supplementary Table 2 *Summary of primary PK parameters for FGF401 single-agent phase 1 part at cycle 1 day 8**

| **Treatment group** | **Cycle 1 day 8** | | | | | | |
| --- | --- | --- | --- | --- | --- | --- | --- |
|  | **AUC_inf_**  **(h*ng/mL)** | **AUC_last_**  **(h*ng/mL)** | **AUC_0-24h_**  **(h*ng/mL)** | **C_max_**  **(ng/mL)** | **T_max_**  **(h)** | **T_1/2_**  **(h)** | **R_acc_** |
| Fasted | | | | | | | |
| 50 mg  (N=10) | n=6 | n=7 | n=7 | n=7 | n=7 | n=6 | n=6 |
|  | 3650  (71.9) | 3460  (62.5) | 3650  (65.6) | 663  (46.8) | 1  [0.5-2] | 6.57  (16.6) | 0.929  (12.1) |
| 80 mg  (N=6) | n=6 | n=6 | n=6 | n=6 | n=6 | n=6 | n=5 |
|  | 5130  (28.9) | 4840  (27.0) | 4850  (27.0) | 838  (16.5) | 1.01  [0.983-2] | 6.08  (13.8) | 0.97  (14.3) |
| 120 mg  (N=26) | n=18 | n=24 | n=24 | n=24 | n=24 | n=18 | n=24 |
|  | 7590  (43.0) | 6480  (47.5) | 6970  (38.7) | 1120  (36.5) | 1.04  [0.533-7.08] | 5.43  (30.0) | 1.03  (26.2) |
| 150 mg  (N=7) | n=6 | n=6 | n=6 | n=6 | n=6 | n=6 | n=6 |
|  | 7850  (29.4) | 7550  (28.6) | 7510  (29.8) | 1070  (46.0) | 2.42  [1-3.42] | 5.24  (12.4) | 0.982  (33.3) |
| Fed | | | | | | | |
| 80 mg  (N=5) | n=4 | n=4 | n=4 | n=4 | n=4 | n=4 | n=4 |
|  | 5550  (23.2) | 5280  (23.9) | 5290  (24.0) | 704  (22.1) | 2.5  [1.17-3.9] | 5.58  (12.0) | 1.04  (18.6) |
| 120 mg  (N=19) | n=15 | n=18 | n=16 | n=18 | n=18 | n=15 | n=15 |
|  | 7980  (25.1) | 7640  (33.1) | 7330  (26.7) | 1060  (22.1) | 3.01  [0.583-6.33] | 5.58  (17.1) | 0.95  (22.5) |
| All the parameters presented as geometric mean (geometric mean CV%), except T_max_, which is presented as median [min, max] | | | | | | | |

*Pharmacokinetic analysis set.

Supplementary Table 3 *Summary of primary PK parameters for spartalizumab in combination arm for cycle 1 day 1**

| **Treatment group** | **Cycle 1 day 1** | | | |
| --- | --- | --- | --- | --- |
|  | **AUC_0-504h_**  **(day*µg/mL)** | **AUC_last_**  **(day*µg/mL)** | **C_max_**  **(µg/mL)** | **T_max_**  **(h)** |
| FGF401 80 mg + spartalizumab 300 mg (N=6) | n=3 | n=6 | n=6 | n=6 |
|  | 760 (1.9) | 795 (34.5) | 74.7 (43.0) | 1.58 [1.5-161] |
| FGF401 120 mg + spartalizumab 300 mg (N=6) | n=6 | n=6 | n=6 | n=6 |
|  | 967 (13.9) | 978 (15.8) | 87.7 (5.4) | 1.5 [1.43-1.5] |
| All the parameters presented as geometric mean (geometric mean CV%), except Tmax, which presented as median [min, max] | | | | |

*Pharmacokinetic analysis set.
