# Supplementary material for: A first-in-human phase 1/2 study of FGF401 and combination of FGF401 with spartalizumab in patients with hepatocellular carcinoma or biomarker-selected solid tumors
Source: J Exp Clin Cancer Res. 2022 Jun 2;41:189. doi: 10.1186/s13046-022-02383-5 (PMC9161616; doi:10.1186/s13046-022-02383-5)
Supplement: Supplementary file 2 — Additional file 2. [file 13046_2022_2383_MOESM2_ESM.docx]

**Supplementary Fig. 1 Arithmetic mean (SD) of (A) FGF401 concentration and (B) spartalizumab concentration in semi-logarithmic view of combination arm at cycle 1 day 1**


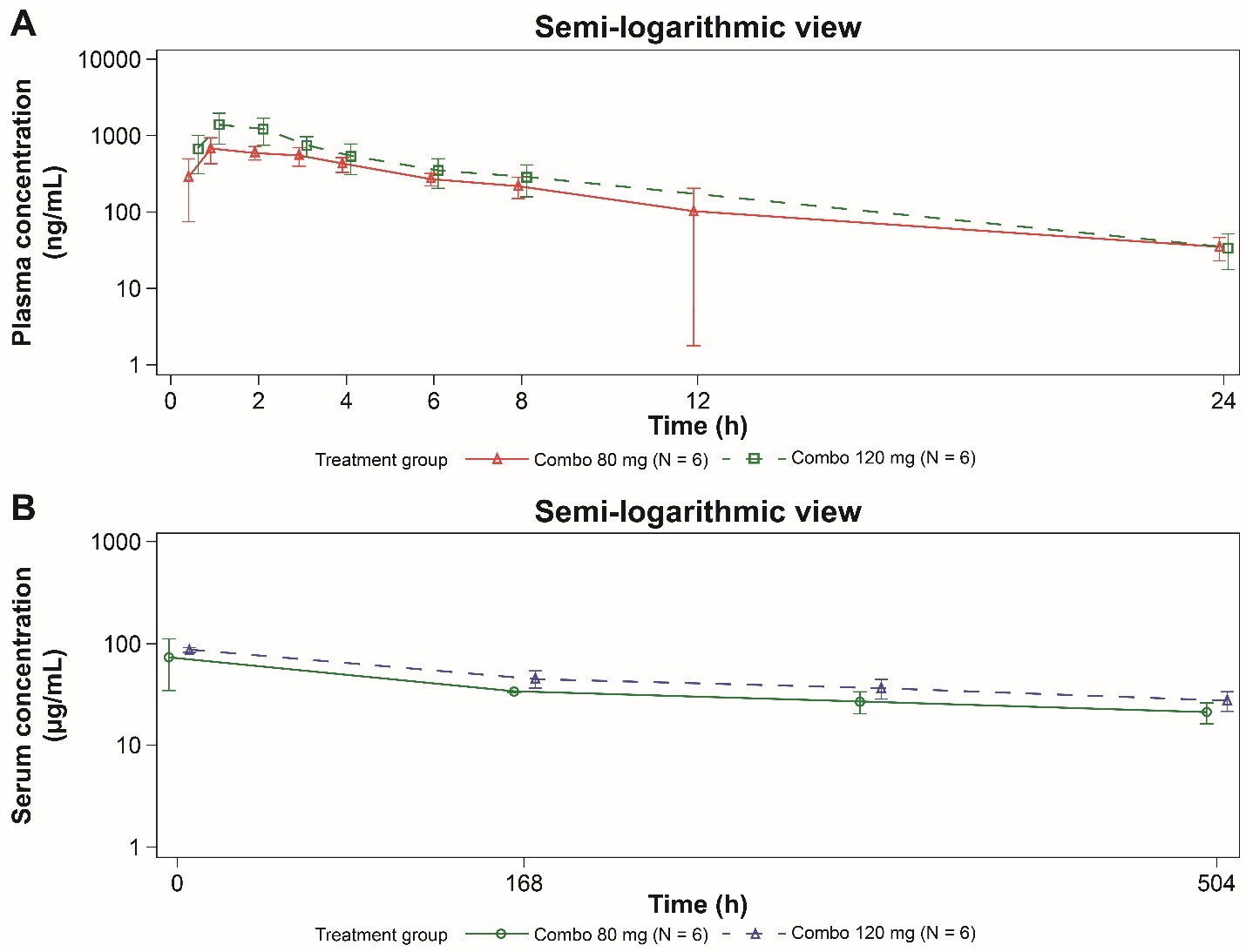


**Supplementary Fig. 2 Response to FGF401.** Waterfall plot for best percentage change from baseline in sum of the longest diameters based on local radiology review per RECIST v1.1; (A) HCC patients with RT‑qPCR–positive expression of FGF19, (B) HCC patients with RT-qPCR–negative expression of FGF19, (C) FGF19 IHC-positive patients with HCC, (D) IHC-negative patients with HCC, (E) FGF19 expression assessed by RT-qPCR and expressed as mean CT versus FGF19 protein expression assessed by IHC. (F) Kaplan-Meier plot of OS in FGF401 single-agent phase 1 RP2D.

For FGF19 RT-qPCR, if CT was ≤35 the sample was considered positive, else negative. Tumors were deemed FGF19+ by IHC when percentage of positive cells >0, regardless of intensity.

CT: cycle threshold.


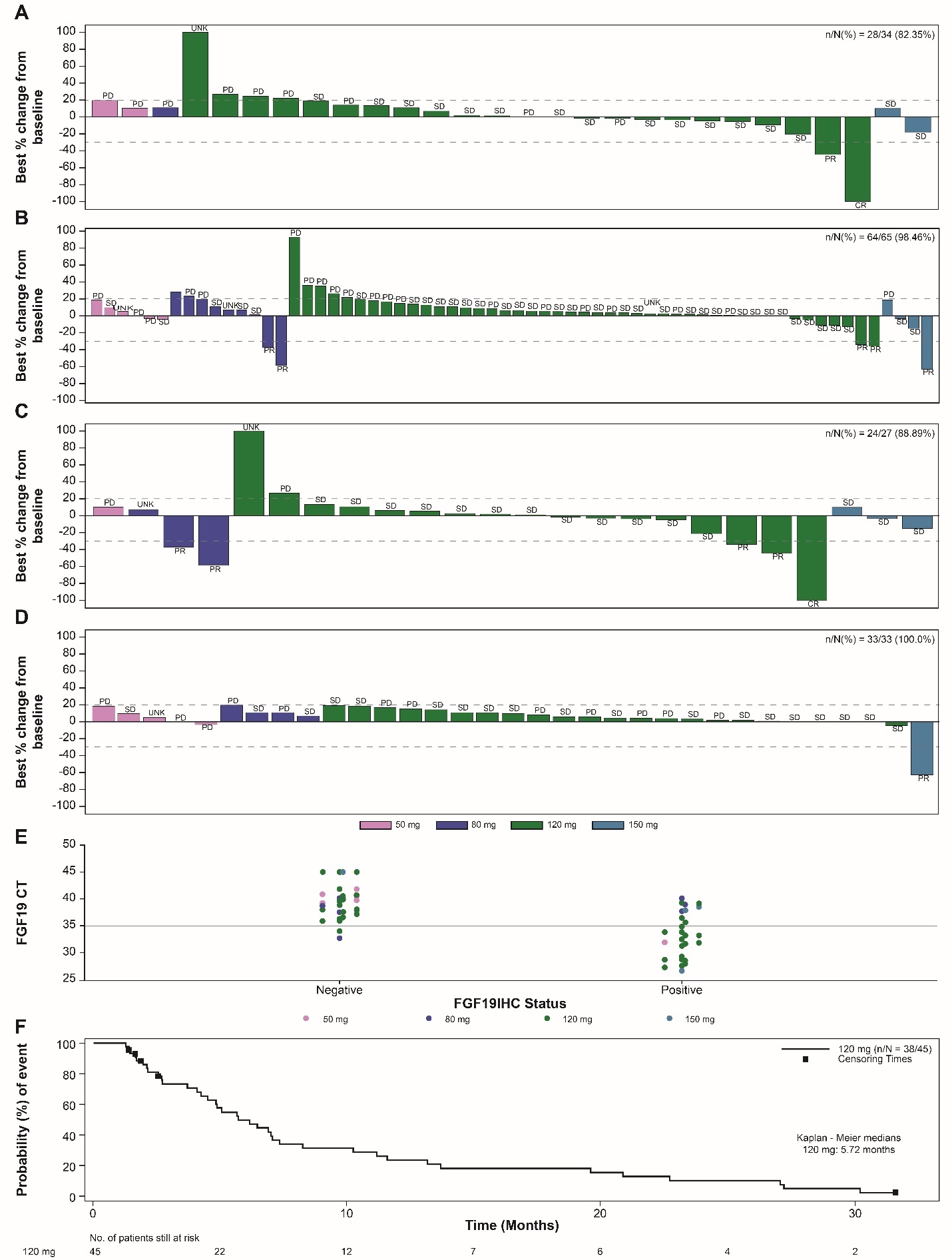


Supplementary Fig. 3 Blood pharmacodynamics and tumor biomarkers modulation in HCC patients treated with FGF401.

A-B. FGF19 and cholesterol levels shown at different days of cycle for the patients with HCC in the 120 mg fasted dose group.

Upon FGFR4 inhibition, (A) circulating FGF19 levels increase and (B) cholesterol levels shown at different cycles decrease with treatment. FGF401 treatment induces (C) elevation of CYP7A transcript levels and (D) suppression of DUSP6 transcript expression in most tumor biopsies at C1D8 (on-treatment) as compared to pre-treatment biopsies.

BL, baseline; ONT, on-treatment.

120mg + 150 mg denotes patients receiving either of 120 mg or 150mg of FGF401. Due to few samples in 150mg cohort, they are represented together with 120mg dose.


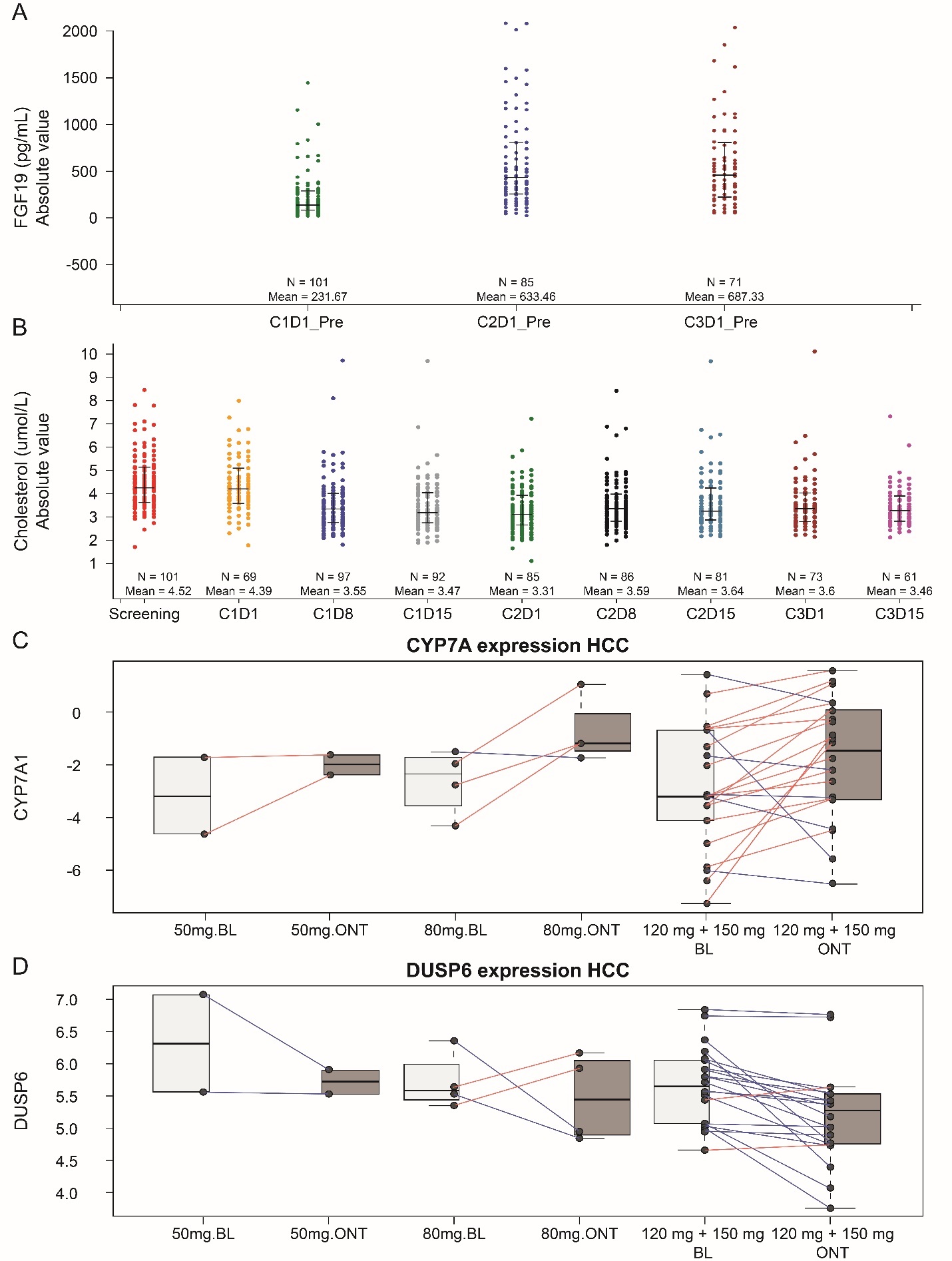


Supplementary Fig. 4 Heatmap showing the expression levels for DUSP6, CYP7A1 and signatures of immune infiltration, summarized with GSVA at screening and on-treatment.
